# Comparison of physical properties of voluntary coughing, huffing and swallowing in healthy subjects

**DOI:** 10.1371/journal.pone.0242810

**Published:** 2020-12-03

**Authors:** Akiko Yawata, Takanori Tsujimura, Ryosuke Takeishi, Jin Magara, Li Yu, Makoto Inoue

**Affiliations:** 1 Division of Dysphagia Rehabilitation, Niigata University Graduate School of Medical and Dental Sciences, Chuo-ku, Niigata, Japan; 2 Department of Respiratory and Critical Care Medicine, Tongji Hospital, Tongji University School of Medicine, Shanghai, China; University of Notre Dame Australia, AUSTRALIA

## Abstract

Coughing, huffing and swallowing protect the airway from aspiration. This study was conducted to compare the physical properties of voluntary coughing, huffing and swallowing in healthy subjects. Ten healthy men were asked to huff, cough and swallow repeatedly. Electromyograms (EMGs) were recorded from the left side of the external oblique (EO), sternocleidomastoid, suprahyoid (SH) and thyrohyoid muscles. Airflow was recorded using a face mask with two-way non-rebreathing valves. The expiratory velocity of huffing and coughing and the SH EMG of all actions presented high intraclass correlation coefficients (> 0.8). The inspiratory and expiratory velocities did not differ significantly between coughing and huffing. The expiratory acceleration of coughing was significantly higher than that of huffing, whereas the expiratory volume of coughing was significantly smaller than that of huffing. The EO EMG of coughing and huffing were significantly larger than that of swallowing. The EO EMG activity during the expiratory phase was significantly higher than that of the other phases of both coughing and huffing. The SH EMG of coughing and huffing were significantly smaller than that of swallowing. Correlation analysis revealed that the expiratory velocity of coughing was strongly positively correlated with that of huffing. The expiratory volume of huffing was significantly positively correlated with hand grip strength. These results suggest that EO and SH muscle activities during huffing or coughing differ those during swallowing, and huffing and coughing may work similarly in expiratory function.

## Introduction

Severe pneumonia is a life-threatening pulmonary disease classified by the site of incidence as either community-acquired pneumonia (CAP) or hospital-acquired pneumonia (HAP). In Japan, pneumonia became the third leading cause of death in 2011, and approximately 96% of patients who died from pneumonia were aged 65 years or older [1]. Marik et al. demonstrated that 5%–15% of CAP cases are aspiration pneumonia [2]. The incidences of aspiration pneumonia for CAP and HAP were reported to exceed 60% and 80%, respectively, in patients hospitalized for pneumonia, and the ratios of aspiration pneumonia increased with age [3]. Aspiration pneumonia is caused by impaired swallowing and coughing, which are referred to as dysphagia and dystussia, respectively [4]. Avoiding aspiration pneumonia is an urgent and important issue.

The voluntary and reflexive cough and swallow protect the airway from aspiration. A forced expiration technique (e.g., huffing) also prevents aspiration because it clears sputum and/or residual food from the airway [5]. Higher expiratory velocity during coughing is associated with a lower risk of pneumonia in acute stroke patients [6]. Shon et al. suggested that measuring cough strength can predict the risk of aspiration pneumonia in patients with dysphagia attributable to cerebrovascular disease or brain lesions [7]. Wakasugi et al. developed a screening system that combined a swallowing test with a coughing test to detect silent aspiration related to the development of aspiration pneumonia in patients with clinical symptoms of dysphagia [8]. These clinical studies emphasize the importance of coughing and swallowing in the incidence of aspiration pneumonia. Furthermore, Park et al. reported that expiratory muscle strength training like huffing increased swallowing-related muscles activity in community-dwelling older subjects [9]. To understand the importance of coughing, huffing and swallowing for preventing the occurrence of aspiration pneumonia in patients, we should know these behaviors in healthy condition as a first step. However, to our knowledge, no studies have clarified how motor activities in coughing, huffing and swallowing are different and related. Thus, we focused on motor activities in coughing, huffing and swallowing in healthy young subjects in the present study.

Physiologically, coughing comprises the inspiratory, compressive and expiratory phases [10,11]. The inspiratory phase involves inhaling air with lengthened expiratory muscles to optimize the length-tension relationship. The compressive phase follows the inspiratory phase and includes a brief glottic closure that allows intrathoracic pressure to build due to isometric contraction of the expiratory muscles. During the expiratory phase, the glottis opens, then a brief burst of expiratory flow is released. Huffing consists of sharp and forced expiration without glottis closure, starting from mid-lung volume to low-lung volume [12]. Swallowing is traditionally subdivided into the oral, pharyngeal, and esophageal phases. The oral phase is considered a transport event in which the food bolus moves from the oral cavity to the oropharynx. The pharyngeal phase comprises a series of reflex movements to transport the food bolus into the esophagus. During the pharyngeal phase of swallowing, the vocal folds are adducted, followed by tongue base movement and hyoid and thyroid elevation with submental muscle activity, vestibular closure, pharyngeal contraction and opening of the upper esophageal sphincter [13–15]. The esophageal phase comprises primary peristalsis of the esophagus. We previously suggested that same airway afferents regulate initiation of both swallowing and coughing [16]. Although coughing, huffing and swallowing likely have a common neural network, little is known about how their physical properties are similar and how they differ. The present study compared the physical properties of coughing, huffing and swallowing in healthy subjects. Hand grip strength is an indicator of physical strength [17]. It has been reported that hand grip strength is related to peak cough flow in old male nursing home residents [18]. In the present study, we also investigate the relation between coughing, huffing and hand grip strength.

## Methods

### Ethical approval

The Ethics Committee of Niigata University (2018–0078) approved this study. Participants were recruited through posters from the Niigata University Faculty of Dentistry. All participants provided written informed consent, and the study was performed in accordance with the Declaration of Helsinki and the Ethical Guidelines for Medical and Health Research Involving Human Subjects. This study is an experimental research design on healthy subjects.

### Electromyography and spirometry

Surface electromyograms (EMGs) were recorded from the left side of the external oblique (EO), sternocleidomastoid (SCM), suprahyoid (SH) and thyrohyoid (TH) muscles. Electrodes (ZB-150H; Nihon Kohden, Japan) were attached to the skin with an interpolar distance of 2 cm and were positioned at the midpoint between the navel and anterior superior iliac spine for the EO muscle, the center of the SCM muscle, the anterior belly of the digastric muscle (one of the SH muscles), and the rostrolateral portion of the thyroid cartilage for the TH muscle [19–21]. Before recording, we confirmed EO muscle activation by having participants bring their left shoulder to their right knee. SCM muscle activation was confirmed by cervical flexure with contralateral rotation, and SH and TH muscle activation was confirmed by voluntary swallowing. Signals were filtered and amplified (low cut: 30 Hz; high cut: 2 kHz) (WEB-1000; Nihon Kohden, Japan). To measure the airflow, participants wore a face mask with two-way non-rebreathing valves connected to the inspiratory and expiratory ports (Oro-Nasal 7400 Vmask, Hans Rudolph Inc., USA). This mask allows measuring the inspiratory and expiratory airflow with separate pathways connected to a spirometer (ML311 Spirometer Pod, AD Instruments, USA). Calibration was performed using a 3L calibration syringe (MLA5530, AD Instruments, USA). The sampling rate was 1 kHz for the EMGs and spirometry. Signals from the EMGs and spirometry were stored on a computer through an interface (PowerLab, AD Instruments, USA).

### Data collection

Subjects were asked not to eat or drink for at least 1 h prior to the experiment. During the recording, subjects were seated comfortably in a chair and wore a mask with EMG electrode placement. After recording the resting respiratory rate for several minutes, subjects were asked to huff, cough and swallow voluntarily at 30-sec intervals in this order as one session. Three sessions were conducted and the time interval between sessions was 1 min. The instructions were as follows: huff (perform a forced expiration at maximum effort after a deep breath), cough (perform a cough at maximum effort after a deep breath), and swallow (perform a saliva swallow). We confirmed huff by without compressive phase which means cessation of airflow. All tasks were recorded by a video to check the body movement.

To evaluate the relationship between airway protective movements and physical strength, dominant hand grip strength was measured using a grip dynamometer (Hydraulic Hand Dynamometer, NC70142, North Coast Medical, Inc., USA).

**Fig 1:**
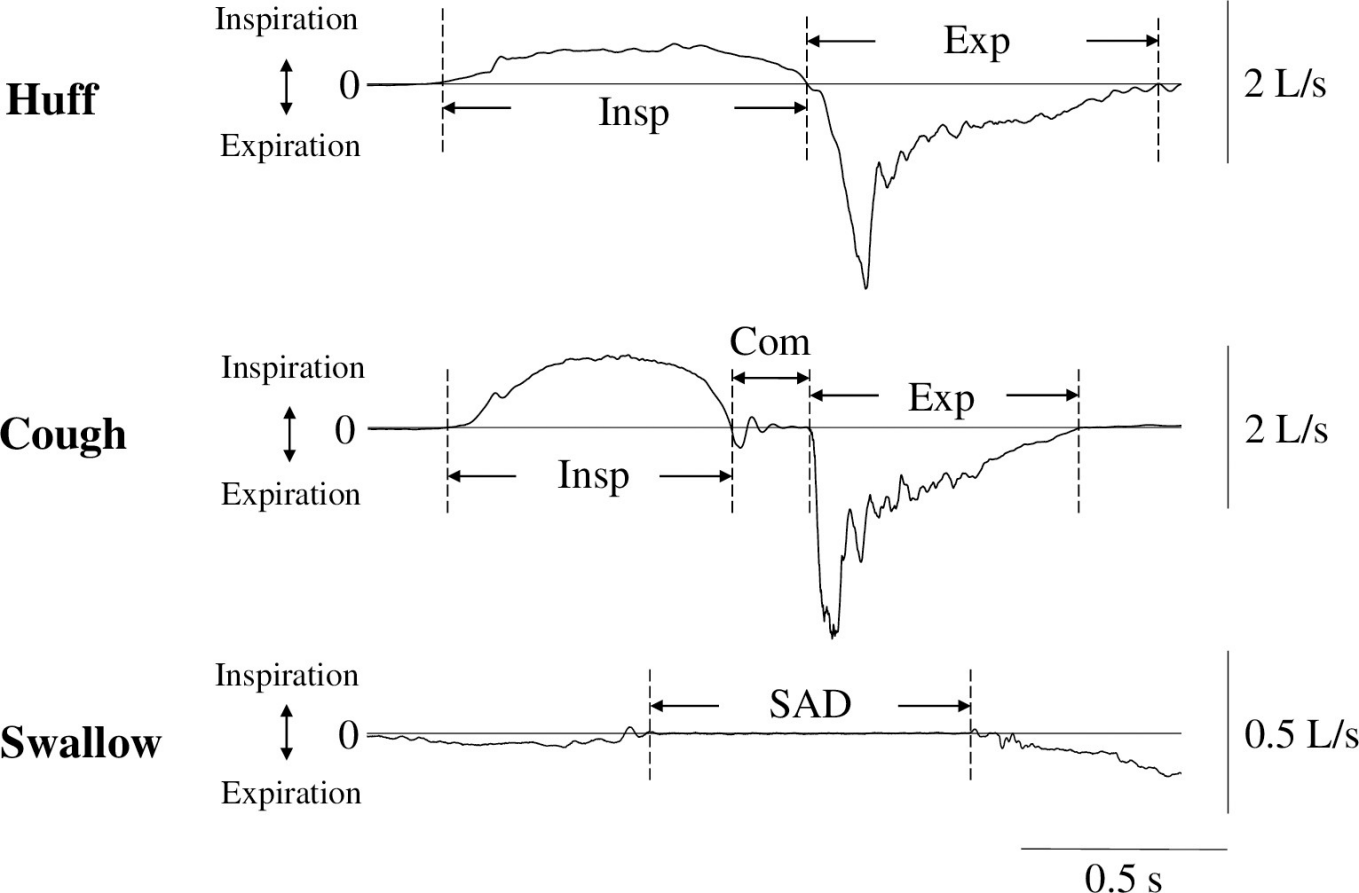
Classification of airflow phases during huffing (Huff), coughing (Cough), and swallowing (Swallow). The recordings were obtained from the same subject. Insp, inspiratory phase duration; Exp, expiratory phase duration; Com, compressive phase duration; SAD, swallowing apnea duration.

Data were synchronized and analyzed using PowerLab software (LabChart8; AD Instruments, USA). The phase was first determined from the airflow changes measured using a pressure transducer connected to a face mask. Fig 1 illustrates the typical airflow during huffing, coughing and swallowing. The inspiratory phase duration was defined as the time between onset and cessation of inspiratory flow during huffing and coughing. The expiratory phase duration was defined as the time between onset and offset of expiratory flow during these actions. Compressive phase duration was defined as the time between cessation of inspiratory flow and onset of expiratory flow during coughing. The swallowing apnea duration was defined as the cessation of airflow during swallowing. Consistent with a previous study, most swallows occurred during the expiration [22]. Peak inspiratory and expiratory velocity were defined as inspiratory and expiratory velocity, respectively, during huffing and coughing. Inspiratory acceleration was calculated by dividing duration from onset to peak in the inspiratory phase into inspiratory velocity. Expiratory acceleration was calculated by dividing duration from onset to peak in the expiratory phase into expiratory velocity. The EMG values were the integral values derived from the area of rectified and smoothed signals [23,24]. Muscle activity was defined as active when the rectified and smoothed EMG signals exceeded the mean + 2 standard deviations of the background activity gained from the 30-sec stable period at rest.

### Data analysis

Except for age, the results are presented as the mean ± standard error of the mean (SEM). We did not statistically determine the sample size, but our sample sizes are comparable to those reported in previous publications [25,26]. Reproducibility of the airflow and EMG parameters was evaluated using the intraclass correlation coefficient (ICC). An ICC > 0.8 is considered to have good reliability [27,28]. Tests for normality and equality of variances were initially performed for statistical analyses to determine whether to use parametric or nonparametric tests. A paired t-test or Wilcoxon’s rank-sum test was used to analyze differences between two groups. One-way repeated-measures analysis of variance or Friedman’s test followed by Tukey’s test were used for multiple comparisons. The correlation coefficient was evaluated using Pearson’s or Spearman’s correlation. Differences were considered significant at p < 0.05.

## Results

### Subjects

Ten healthy men (mean age ± SD: 30.6 ± 4.8 years, range: 25–40 years) participated in this study. Inclusion criteria were an age of 20 years or older, nonsmoker and no coughing, chewing, swallowing and speech problems. The subjects with histories of respiratory or neurological disease were excluded.

### Characterization of airflow and EMGs of huffing, coughing and swallowing

The mean respiratory rate at rest was 13.3 ± 1.2 cycle/min (n = 10), which was considered to be within the normal range, suggesting that the respiratory function at rest was less influenced under this experimental condition.

**Fig 2:**
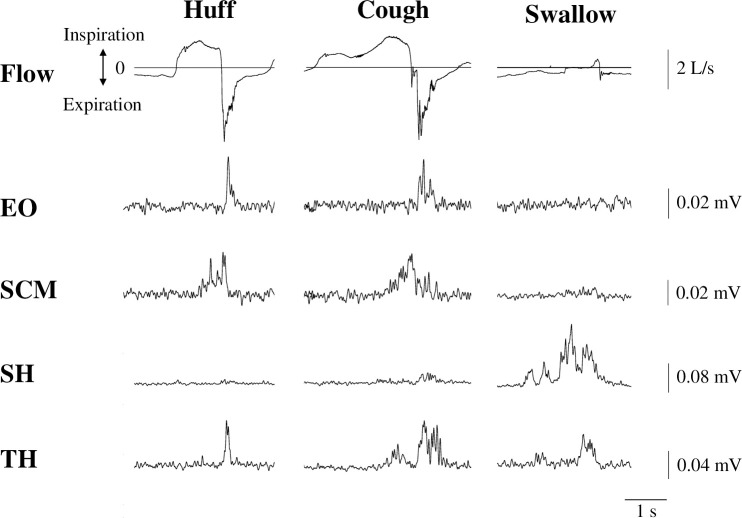
Representative electromyogram (EMG) lines showing huffing (Huff), coughing (Cough), and swallowing (Swallow). Airflow (Flow) and EMG lines during huffing (left), coughing (middle) and swallowing (right). The recordings were obtained from the same subject. EO, external oblique muscle; SCM, sternocleidomastoid muscle; SH, suprahyoid muscles; TH, thyrohyoid muscle.

Fig 2 illustrates the typical airflow and the EO, SCM, SH and TH EMGs during huffing, coughing and swallowing. During huffing, deep inspiration occurred with SCM EMG activity, followed by a sharp expiration with EO, SCM and TH EMG burst. During coughing, deep inspiration occurred with SCM and small TH EMG burst, followed by cessation of inspiratory flow with SCM, TH and small SH EMG burst, and finally a very sharp expiration with EO, SCM, TH and small SH EMG burst. Huffing and coughing differ in the existence of a compression phase in which the glottis closes. During swallowing, SH EMG activity occurred, followed by airflow cessation with SH, TH and small SCM EMG activity.

### Reproducibility of airflow and EMG parameters

First, we investigated the reproducibility of the airflow and EMG parameters of huffing, coughing and swallowing. Because we failed to record huffing for one participant in the first trial, the huffing results were analyzed for nine participants. The means (range) for inspiratory and expiratory duration of huffing were 1.63 s (0.63–3.92 s) and 1.10 s (0.19–2.68 s), respectively; those for the inspiratory, compressive and expiratory durations of coughing were 1.18 s (0.63–2.17 s), 0.267 s (0.036–1.015 s) and 1.01 s (0.30–2.71 s), respectively. The swallowing apnea duration was 0.652 s (0.327–0.970 s). The durations from onset to peak in the inspiratory and expiratory phases during huffing were 1.02 s (0.06–3.14 s) and 0.110 s (0.050–0.279 s), respectively, and those in the inspiratory and expiratory phases during coughing were 0.754 s (0.113–1.927 s) and 0.0486 s (0.0060–0.1800 s), respectively. The ICCs for all duration parameters were < 0.8 (Table 1). These results suggest that the phase duration parameters varied.

**Table 1:** Reproducibility of airflow and EMG parameters of huffing (Huff), coughing (Cough) and swallowing (Swallow).

Task	Duration						Velocity				Volume		EMG			
	Insp	Com	Exp	InspPeak	ExpPeak	SAD	Insp	Exp	InspAcc	ExpAcc	Insp	Exp	EO	SCM	SH	TH
Huff	0.026	NA	0.631	-0.055	0.256	NA	0.307	**0.931**	-0.135	**0.870**	0.243	**0.881**	0.783	**0.845**	**0.842**	0.421
Cough	0.130	0.655	0.287	0.235	0.340	NA	0.514	**0.807**	-0.062	0.468	0.658	0.648	0.718	**0.845**	**0.847**	0.520
Swallow	NA	NA	NA	NA	NA	0.504	NA	NA	NA	NA	NA	NA	NA	0.779	**0.823**	**0.934**

Intra-individual reproducibility for each parameter was evaluated using the intraclass correlation coefficient. n = 9 for huffing and 10 for coughing and swallowing. NA, not applicable; Insp, inspiratory phase; Com, compressive phase; Exp, expiratory phase; InspPeak, duration from onset to peak in inspiratory phase; ExpPeak, duration from the onset to peak in expiratory phase; SAD, swallowing apnea duration; InspACC, inspiratory acceleration; EO, external oblique muscle.

The inspiratory and expiratory velocities were 1.92 L/s (1.12–2.97 L/s) and 5.45 L/s (1.70–10.42 L/s) during huffing and 2.21 L/s (1.26–3.70 L/s) and 5.83 L/s (2.79–12.44 L/s) during coughing, respectively. The inspiratory and expiratory accelerations of huffing were 3.83 L/s^2^ (0.73–23.83 L/s^2^) and 59.1 L/s^2^ (8.4–116.5 L/s^2^), respectively; those of coughing were 4.31 L/s^2^ (1.15–28.77 L/s^2^) and 201 L/s^2^ (27–749 L/s^2^), respectively. The inspiratory and expiratory volumes were 1.99 L (0.71–5.82 L) and 1.59 L (0.53–3.69 L) during huffing and 1.35 L (0.53–2.90 L) and 1.08 L (0.38–3.25 L) during coughing, respectively. The ICCs for expiratory velocity of huffing and coughing and expiratory acceleration and volume of huffing were all > 0.8, suggesting that these parameters had high intra-individual reproducibility (Table 1). The SH EMG of all actions, the SCM EMG of huffing and coughing, and the TH EMG of swallowing also showed high ICCs (Table 1). EO EMG was not observed during swallowing. Multiple comparison revealed that there were no significant difference among first, second and third trials in all parameters with < 0.8 ICC. Although some parameters showed quite variable, we confirmed that trial order did not affect the results. Because we failed to record huffing for one subject in the first trial, we used the data obtained from the second trial for all participants in the following analysis.

### Comparison of airflow parameters between huffing and coughing

**Fig 3:**
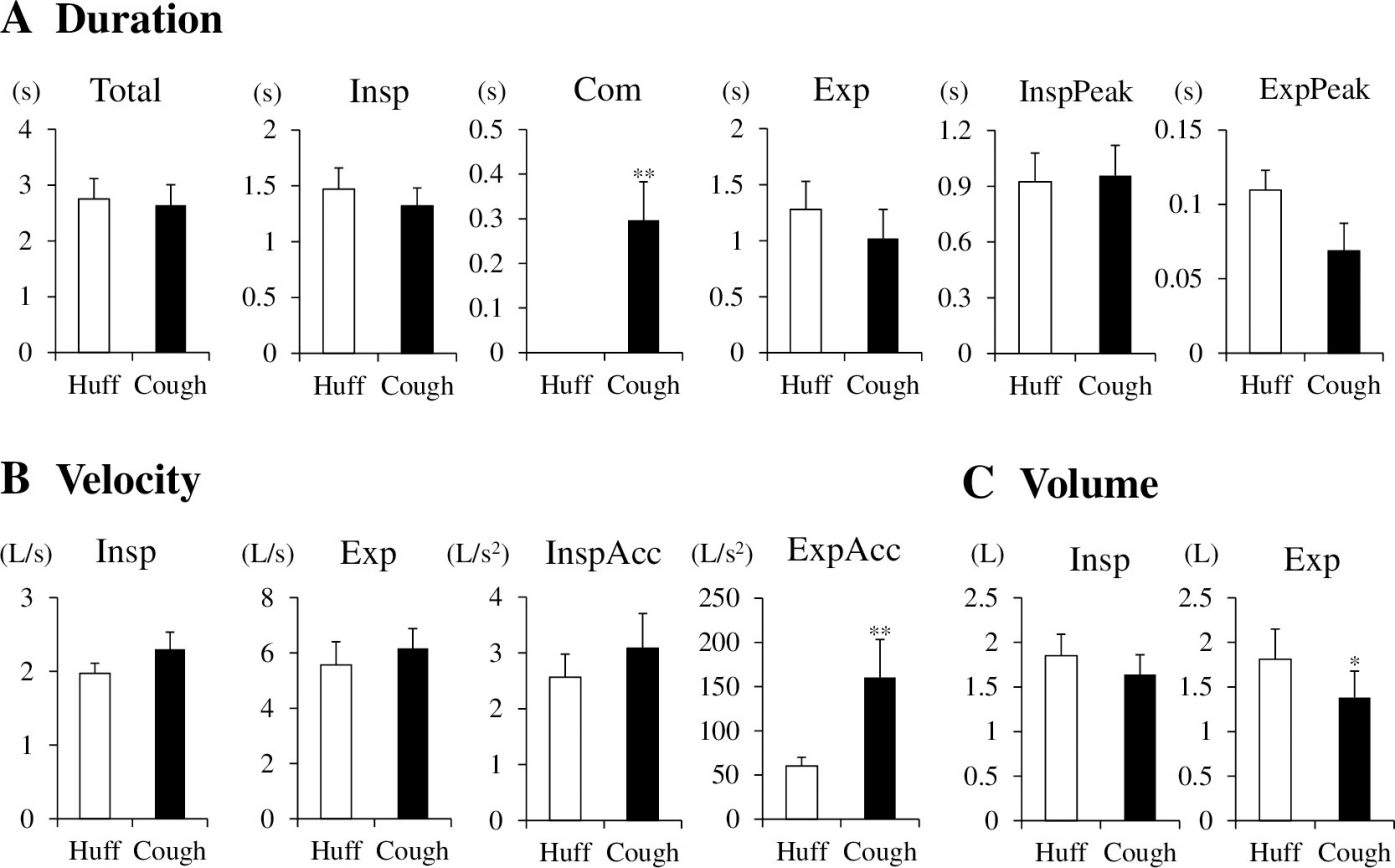
Comparison of airflow parameters between huffing (Huff) and coughing (Cough). n = 10 per group. **p < 0.01 vs Huff, *p < 0.05 vs Huff. Paired t-test or Wilcoxon’s rank-sum test. Total, total duration; Insp, inspiratory phase; Com, compressive phase; Exp, expiratory phase; InspPeak, duration from onset to peak in the inspiratory phase; ExpPeak, duration from onset to peak in the expiratory phase; InspAcc, inspiratory acceleration; ExpAcc, expiratory acceleration.

We compared the duration, velocity and volume between huffing and coughing (Fig 3). Except for the compressive phase duration, huffing and coughing did not significantly differ among the duration parameters (Fig 3A, Total: *p* = 0.705, Insp: *p* = 0.393, Com: *p* = 0.002, Exp: *p* = 0.229, InspPeak: *p* = 0.802, ExpPeak: *p* = 0.069). The inspiratory and expiratory velocities did not significantly differ between huffing and coughing (Insp: *p* = 0.102, Exp: *p* = 0.339). Expiratory acceleration during coughing was significantly higher than that during huffing, whereas inspiratory acceleration did not differ between coughing and huffing (Fig 3B, InspAcc: *p* = 0.370, ExpAcc: *p* = 0.006). Because expiratory velocity was similar between huffing and coughing, the difference in expiratory acceleration was likely due to the difference in duration from onset to peak in the expiratory phase. The duration from onset to peak in the expiratory phase during coughing tended to be shorter than that during huffing (*p* = 0.069). Although the inspiratory volumes did not significantly differ between the two tasks, expiratory volume during coughing was significantly lower than that during huffing (Fig 3C, Insp: *p* = 0.250, Exp: *p* = 0.038). The residual air volume was likely located inside the airway after coughing compared with that after huffing.

### Comparison of EMG parameters of huffing, coughing and swallowing

**Fig 4:**
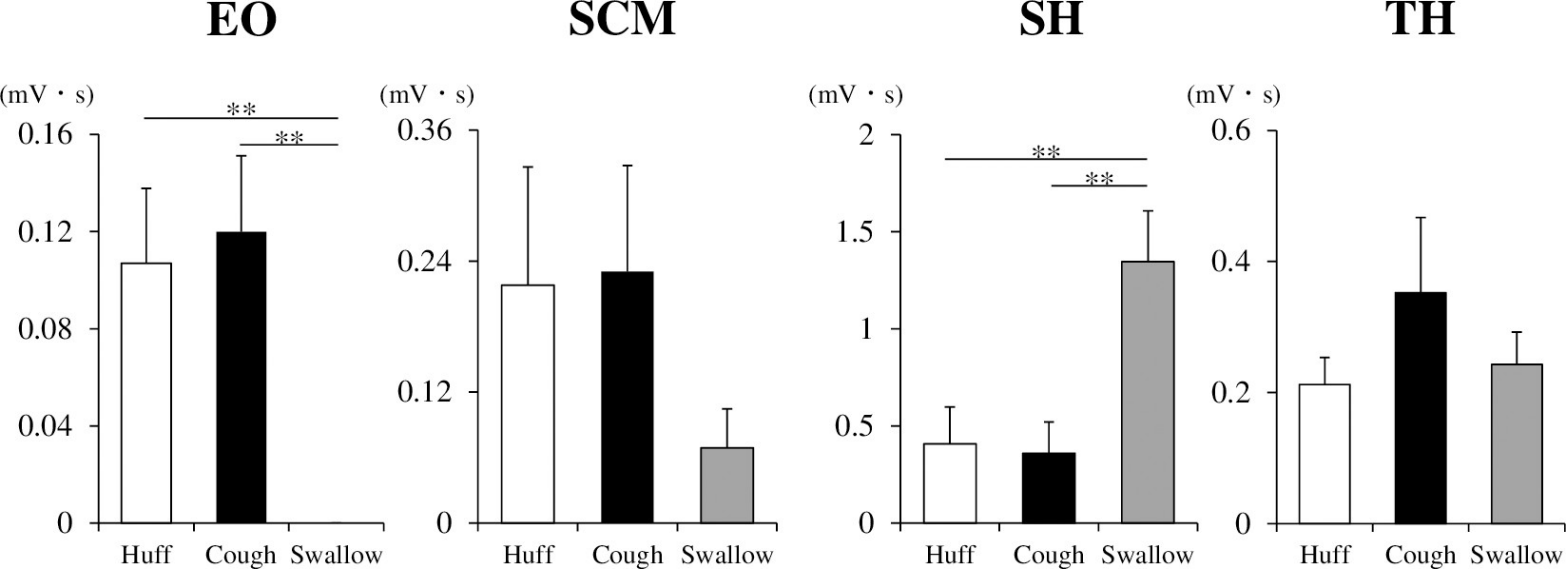
Comparison of EMG activities during huffing (Huff), coughing (Cough) and swallowing (Swallow). n = 10 per group. **p < 0.01. One-way repeated-measures analysis of variance or Friedman’s test followed by Tukey’s test. EO, external oblique muscle; SCM, sternocleidomastoid muscle; SH, suprahyoid muscles; TH, thyrohyoid muscle.

We compared the EO, SCM, SH and TH EMG activities of huffing, coughing and swallowing (Fig 4). The EO EMG activity of coughing and huffing did not differ from each other and were significantly larger than that of swallowing (*F* (2, 9) = 11.823, *p* < 0.001, post hoc Tukey test; Huff vs Cough: *p* = 0.882, Huff vs Swallow: *p* = 0.003, Cough vs Swallow: *p* = 0.001). The SCM EMG activity did not significantly differ among these actions (*F* (2, 9) = 0.788, *p* = 0.47). The SH EMG activity of coughing and huffing were not different from each other and were significantly smaller than that of swallowing (*F* (2, 9) = 11.049, *p* < 0.001, post hoc Tukey test; Huff vs Cough: *p* = 0.977, Huff vs Swallow: *p* = 0.003, Cough vs Swallow: *p* = 0.002). The TH EMG activity did not significantly differ among these actions (Friedman’s test; *p* = 0.497).

**Fig 5:**
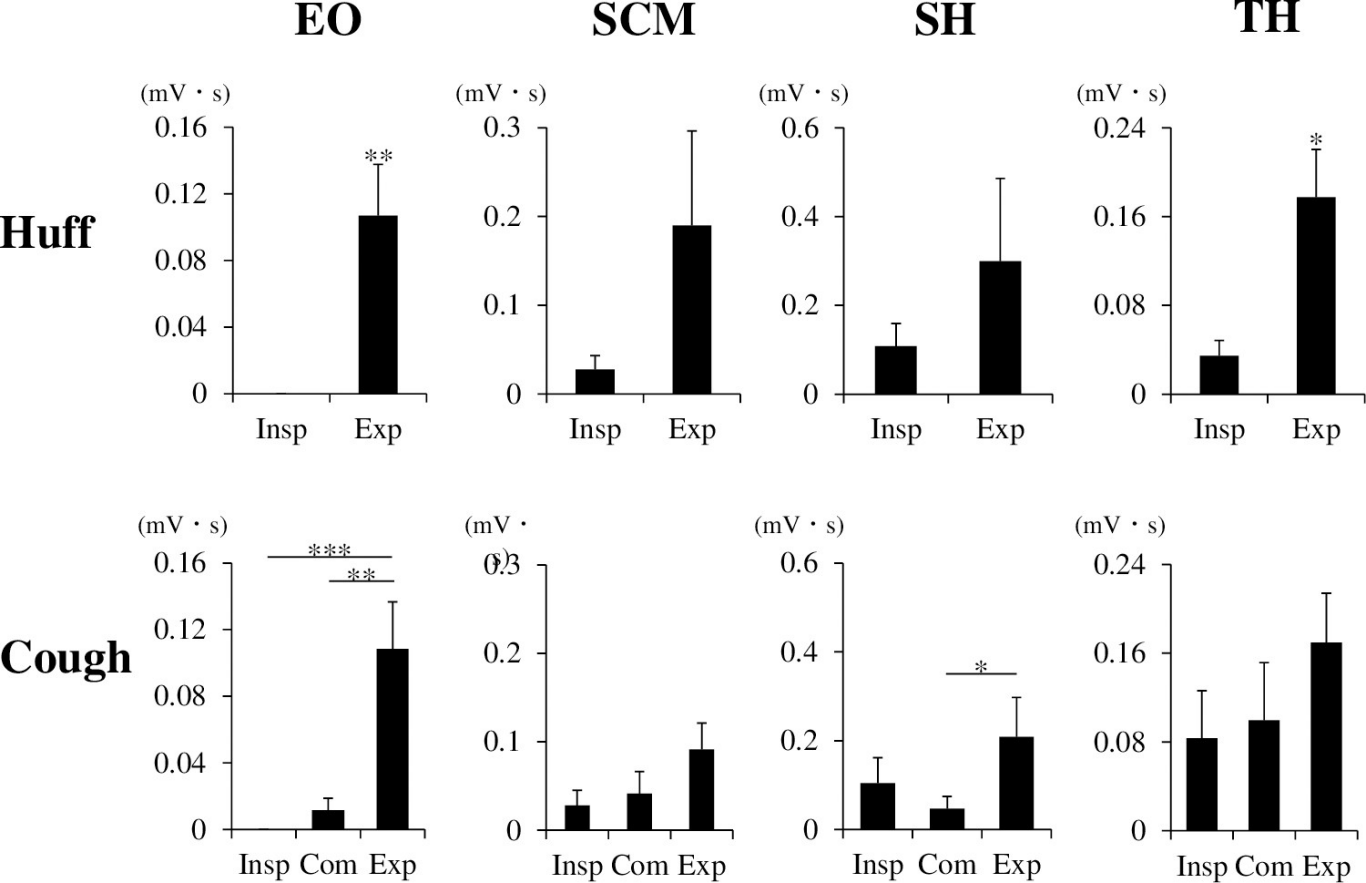
Comparison of EMG activities among airflow phases during huffing (Huff) and coughing (Cough). n = 10 per group. ***p < 0.001, **p < 0.01, *p < 0.05. Paired t-test or Wilcoxon’s rank-sum test for huffing. One-way repeated-measures analysis of variance or Friedman’s test followed by Tukey’s test for coughing. EO, external oblique muscle; SCM, sternocleidomastoid muscle; SH, suprahyoid muscles; TH, thyrohyoid muscle; Insp, inspiratory phase; Com, compressive phase; Exp, expiratory phase.

We further analyzed the EMG parameters of coughing and huffing to discriminate among airflow phases (Fig 5). The EO EMG activity of huffing and coughing were significantly larger during the expiratory phase than during the other phases (Huff: *p* = 0.007, Cough: *F* (2, 9) = 13.515, *p* < 0.001, post hoc Tukey test; Insp vs Com: *p* = 0.710, Insp vs Exp: *p* < 0.001, Com vs Exp: *p* = 0.002). The SCM EMG activity did not significantly differ among the phases (Huff: *p* = 0.156, Cough: Friedman’s test; *p* = 0.050). The SH EMG activity during the expiratory phase of coughing was significantly larger than that during the compressive phase (Huff: *p* = 0.844, Cough: Friedman’s test; *p* = 0.010, post hoc Tukey test; Insp vs Com: *p* = 0.973, Insp vs Exp: *p* = 0.065, Com vs Exp: *p* = 0.037). The TH EMG activity during the expiratory phase of huffing was significantly larger than that during the inspiratory phase (Huff: *p* = 0.016, Cough: Friedman’s test; *p* = 0.061).

### Relationships among airflow parameters and grip strength

We evaluated the correlation between airflow parameters with ICC > 0.8 and grip strength (Table 2). EMG parameters were excluded to consider the influence of different baseline conditions among subjects. All participants were right-handed. Dominant hand grip strength was measured using a grip dynamometer (mean ± SEM: 41.6 ± 1.9, range: 33–52 kg, n = 10). The expiratory velocity of huffing was strongly positively correlated with that of coughing (*p* < 0.01). The expiratory acceleration of huffing was strongly positively correlated with expiratory volume of huffing (*p* < 0.01). The expiratory volume of huffing was significantly positively correlated with hand grip strength (*p* < 0.05).

**Table 2:** Correlation among airflow parameters and grip strength.

	1	2	3	4	5
1. Huff Expiratory Velocity	1				
2. Huff Expiratory Acceleration	.609	1			
3. Huff Expiratory Volume	.558	.773**	1		
4. Cough Expiratory Velocity	.879**	.560	.540	1	
6. Grip	-.036	.565	.666*	-.078	1

Correlation among airflow parameters and grip strength. n = 10 per group. **p < 0.01, *p < 0.05. Pearson’s or Spearman’s correlation. Huff Expiratory Velocity, expiratory velocity of huffing; Huff Expiratory Acceleration, expiratory acceleration of huffing; Huff Expiratory Volume, expiratory volume of huffing; Cough Expiratory Velocity, expiratory velocity of coughing; Grip, hand grip strength.

## Discussion

We investigated the physical properties of voluntary coughing, huffing and swallowing in healthy men. The expiratory velocity and SCM EMG of coughing and huffing, TH EMG of swallowing, and SH EMG of all actions showed high reproducibility. The inspiratory and expiratory velocities did not differ between coughing and huffing. Expiratory acceleration of coughing was significantly higher than that of huffing, while the expiratory volume of coughing was significantly smaller than that of huffing. The EO EMG activity of coughing and huffing were significantly larger than that of swallowing. The SH EMG activity of coughing and huffing were significantly smaller than that of swallowing. The expiratory velocity of coughing was strongly positively correlated with that of huffing. The expiratory volume of huffing was significantly positively correlated with hand grip strength. These results suggest that EO and SH muscle activities during huffing or coughing differ those during swallowing, and huffing and coughing may work similarly in expiratory function.

A clinical study showed that peak expiratory velocity of huffing was significantly smaller than that of coughing in patients with airways obstruction [29]. Because inspiratory volume is the most important determinant of expiratory velocity of voluntary coughing in healthy subjects [30] and huffing is starting from approximately mid-lung volume [29], the difference in peak expiratory velocity between coughing and huffing in the previous study was likely due to the different inspiratory volume. To eliminate this influence between coughing and huffing when comparing their maximum ability, we instructed subjects to inhale as deeply as possible for both coughing and huffing in this study. Our results showed that expiratory velocity did not significantly differ between coughing and huffing. The major difference between coughing and huffing is that coughing includes a compressive phase, meaning that the glottis is closed. Previous studies have suggested the importance of the compressive phase on motor activity of coughing [31,32]. Notably, huffing showed a similar expiratory velocity to that of coughing under the instruction of maximum inhalation. The instructions for how to cough and huff might limit the interpretations of this work. Several studies have demonstrated that cough can be significantly affected by instructions. We consider the high correlation between the expiratory effort between cough and huff under the instructions to produce a maximal behavior and it is difficult to conclude that this is the characteristic of these behaviors themselves.

Although the ICC for expiratory velocity of coughing and huffing showed high reproducibility, the ICC for huffing was higher than that for coughing. Furthermore, expiratory volume and expiratory acceleration showed high reproducibility for huffing but not for coughing, suggesting that expiratory airflow parameters are steadier for huffing than for coughing, although all duration parameters of both coughing and huffing varied. The expiratory velocity of huffing and coughing were strongly positively correlated. The EMG data showed no significant difference between coughing and huffing. Furthermore, the EO EMG during the expiratory phase was larger than that during the inspiratory phase of both coughing and huffing. Thus, we speculate that huffing and coughing following maximum inhalation may work similarly in expiratory function.

The SH EMG showed high reproducibility among all actions and was larger of swallowing than of coughing and huffing. Considering the attached position of the electrode, we speculated that the SH EMG activity were recorded from anterior belly of the digastric muscle, mylohyoid and geniohyoid muscles, all of which run from the mandible to the hyoid bone [33]. We expected SH activity is critical for swallowing than that huffing and coughing with consideration of their anatomical locations and functions. Consistent with our hypothesis, SH EMG activity in swallowing was larger than that in huffing and coughing. Ertekin et al. described the importance of SH muscle activity of voluntary swallowing [34]. The SH EMG during the expiratory phase of coughing and huffing tended to be larger than that during the inspiratory phase. Although SH muscles are involved in both jaw opening and hyoid elevation, the role of these muscles in coughing and huffing remains unclear and should be investigated in future studies. A recent clinical study showed that expiratory muscle strength training, when set at 70% maximum expiratory pressure following maximum inhalation, increased SH muscles activity of swallowing in older subjects [9]. These results indicate that the SH muscles may have related functions between huffing and swallowing. TH EMG showed high ICC for swallowing but not for huffing or coughing. The TH muscle arises from the thyroid cartilage and inserted into the inferior aspect of the hyoid bone and is involved in anterior laryngeal excursion and anteroposterior UES opening during swallowing [33]. The high reproducibility of the TH muscle activity during swallowing may have been less voluntary movement because this muscle is mainly involved in reflex movement during swallowing, i.e., laryngeal elevation. Although we expected TH EMG activity in swallowing is larger than that in huffing and coughing, TH EMG activity was not significantly different among these three behaviors. In huffing, TH EMG was significantly larger during the expiratory phase than during the inspiratory phase, indicating that the TH muscle works mainly during the expiratory phase of huffing. EO muscle is one of the abdominal muscles and originates on the external surface of fifth-twelfth ribs and inserts on linea alba, pubic tubercle and anterior half of liliac crest. EO muscle compresses and supports abdominal viscera, and flexes and rotates trunk [35]. As we expected, the EO EMG of coughing and huffing were larger than that of swallowing. A previous study suggested that EO muscle activity is important for expiratory velocity of coughing in healthy subjects [36]. Consistent with this finding, we showed that EO EMG during the expiratory phase of coughing and huffing was larger than that during the other phases. These results suggest that EO and SH muscles of coughing and huffing may work similarly but differently compared with those of swallowing. Because the SCM muscle is an accessory inspiratory muscle, we expected the SCM EMG activity of coughing and huffing are larger than that of swallowing. However, this muscle activity was not significantly different among three behaviors. We detected SCM muscle activation in both the inspiratory and expiratory phases. SCM muscle originates on anterior surface of manubrium and upper surface of medial 1/3 of clavicle and inserts on lateral surface of mastoid process and lateral half of superior nuchal line of occipital bone [35]. The SCM muscle contributes to neck and head flexion and raising thorax [35,37]. In this study, we asked subjects to cough and huff with free positioning of the head to reduce voluntary control as much as possible under limited instruction. Although we did not measure the head position or neck movement during coughing and huffing, all subjects showed neck flexion in accordance with exhalation. We speculate that this is why SCM muscle activity was detected during expiratory phase.

Correlation analysis revealed that expiratory volume of huffing was positively correlated with hand grip strength. Consistent with our results, some previous studies showed that expiratory volume referred as forced vital capacity was positively correlated with hand grip strength in healthy subjects [38,39]. On the other hand, the expiratory velocity of huffing and coughing were not significantly correlated with hand grip strength in the present study. Lavietes et al. hypothesized that determination factors of cough flow are lung recoil and airway resistance [40]. Because lung recoil is dependent on inspiratory muscles contraction and is related to expiratory volume which was significant positive correlated with hand grip strength, the difference of airway resistance among subjects might affect the relation between expiratory velocity and hand grip strength.

We have to consider four methodological limitations. First, we recorded just two respiratory muscles, i.e. EO and SCM, in this study. It would be difficult to discern the complex muscle activities by just recording the two muscles for each function. In consideration the number of other accessory muscles, a multichannel EMG recording would have been ideal in the future study. Second, only males were recruited in this study. Because different respiratory muscle characteristics between male and female are suggested [41], female data should have been provided in the future study. Third, we detected airflow from the mouth through a face mask. Because the trachea, larynx and pharynx are critical areas for sputum clearance, further experiments are needed to measure the airflow at these regions with laryngeal muscles EMG. Fourth, no sample size calculation in the planning and execution of this project is a matter of concern as it limits the extrapolation of findings.

## Conclusion

This study was conducted to compare the physical properties of voluntary coughing, huffing and swallowing in healthy subjects. The inspiratory and expiratory velocities did not differ significantly between coughing and huffing. The EO EMG of coughing and huffing were significantly larger than that of swallowing The SH EMG of coughing and huffing were significantly smaller than that of swallowing. These results suggest that EO and SH muscle activities during huffing or coughing differ those during swallowing, and huffing and coughing may work similarly in expiratory function. In the future study, we should investigate the motor mechanics of airway protective behaviors in older subjects and patients with dysphagia and dystussia to develop an effective approach to avoid aspiration pneumonia.
